# Expression, Regulation, and Functions of the Galectin-16 Gene in Human Cells and Tissues

**DOI:** 10.3390/biom11121909

**Published:** 2021-12-20

**Authors:** Jennifer D. Kaminker, Alexander V. Timoshenko

**Affiliations:** Department of Biology, The University of Western Ontario, London, ON N6A 5B7, Canada; jkamink@uwo.ca

**Keywords:** galectin, *LGALS16*, placenta, brain tissues, cell differentiation, transcription factor, miRNA

## Abstract

Galectins comprise a family of soluble β-galactoside-binding proteins, which regulate a variety of key biological processes including cell growth, differentiation, survival, and death. This paper aims to address the current knowledge on the unique properties, regulation, and expression of the galectin-16 gene (*LGALS16*) in human cells and tissues. To date, there are limited studies on this galectin, with most focusing on its tissue specificity to the placenta. Here, we report the expression and 8-Br-cAMP-induced upregulation of *LGALS16* in two placental cell lines (BeWo and JEG-3) in the context of trophoblastic differentiation. In addition, we provide the results of a bioinformatics search for *LGALS16* using datasets available at GEO, Human Protein Atlas, and prediction tools for relevant transcription factors and miRNAs. Our findings indicate that *LGALS16* is detected by microarrays in diverse human cells/tissues and alters expression in association with cancer, diabetes, and brain diseases. Molecular mechanisms of the transcriptional and post-transcriptional regulation of *LGALS16* are also discussed based on the available bioinformatics resources.

## 1. Introduction

Galectins comprise a family of soluble β-galactoside binding proteins, which regulate key biological processes including cell growth, differentiation, apoptosis, and immune responses [1,2,3,4]. Sixteen galectin genes have been identified in animal kingdoms, 12 of which are expressed in humans. Galectins share a conserved carbohydrate recognition domain (CRD) and they are subcategorized into prototype, tandem-repeat, or chimeric type according to their number of CRDs and structural features. Prototype galectins contain one CRD and include galectins -1, -2, -5, -7, -10, -11, -13, -14, -15, and -16. Tandem-repeat galectins contain two homologous CRDs connected by a linker of ~70 amino acids and include galectins-4, -6, -8, -9, and -12. The only chimera-type galectin is galectin-3, which contains one CRD linked to a non-lectin N-terminal proline/glycine-rich domain. Galectins form a network of proteins to perform glycan-dependent and glycan-independent functions both intra- and extracellularly [3,4,5]. Intracellularly, galectins have multiple binding partners and primarily function via glycan-independent mechanisms to regulate processes such as cell growth, apoptosis, and pre-mRNA splicing among others [4,5,6]. Extracellular galectins are secreted from cells through unconventional mechanisms [3,7] and can bind to glycoligands on the cell surface or glycoproteins in the extracellular matrix to promote cell adhesion and migration [8] or bind to specific cell surface receptors to facilitate their cross-linking and transmembrane signaling [3,8,9,10]. 

Galectin expression profiles vary significantly between different cells and tissues. Some galectins are commonly expressed with low tissue specificity, e.g., galectin-1 and galectin-3, while others are highly-tissue specific [3]. *LGALS16* was characterized in placental tissue by Than and co-authors [11] and together with two other galectins (*LGALS13* and *LGALS14*) was found to be upregulated in differentiated trophoblast cells to confer immunotolerance at the maternal–fetal interface [12]. These three galectin genes are located in a cluster of four human protein-coding galectin genes on chromosome 19 and they are proposed to have evolutionarily emerged to sustain hemochorial placentation in anthropoids [11]. The correct expression of placenta-specific galectins is an important part of proper reprogramming of the transcriptional activity of the trophoblast [12]. This involves the differentiation and fusion of villous cytotrophoblasts into a multinucleated syncytium that is in direct contact with maternal blood and is responsible for facilitating gas, nutrient, and waste exchange between the mother and fetus, mediating hormonal regulation, and forming an immunological barrier during pregnancy [12]. Differentiated extravillous trophoblasts proliferate, invade, and remodel the maternal spiral arteries to provide blood flow and nutrients to the fetus [13]. Dysregulation of this placenta-specific gene cluster containing *LGALS16* is associated with disorders such as preeclampsia, which can be highly fatal for both the mother and fetus [12,13,14]. 

Currently, experimental studies on *LGALS16* are limited, although multiple microarray datasets and bioinformatics resources contain relevant information. Here, we use experimental and bioinformatics approaches for examining expression, regulation, and functions of *LGALS16* to position this galectin within the complex galectin network in cells and to identify directions for future studies.

## 2. Materials and Methods

### 2.1. Bioinformatics Data and Tools

Microarray and RNA-sequencing data were extracted from the Gene Expression Omnibus (GEO) Profiles, which contained 287 datasets for *LGALS16* (accessed on 2 November 2021), considering the following criteria: (1) inclusion of only controls and untreated cell/tissue samples, (2) inclusion of only cases with positive gene expression values for matched *ACTB* (a housekeeping gene), *LGALS1* (a low tissue specific galectin), and *LGALS16* genes, and (3) deletion of few datasets, which report enormous deviations (>100-folds) from average expression levels of *ACTB* and *LGALS1* genes. In silico prediction of transcription factor binding sites in *LGALS16* gene DNA sequence was performed with PROMO version 3.0.2 software, which utilized version 8.3 of TRANSFAC database [15,16]. The dissimilarity index for the transcription factor search was set at 0% to limit the number of non-specific matches. Ensembl Release 104 was used to extract the sequence of the 2 kb promoter region of the gene (accessed on 24 August 2021). Four different online platforms were used and compared to predict putative miRNA targets for *LGALS16* including Diana Tools [17], miRabel [18], miRDB [19], and TargetScan [20]. The Human Protein Atlas (HPA) [21], GenBank [22], and Protein Data Bank (PDB) [23] were exploited for searching the relevant structures, sequences, and expression patterns of *LGALS16* based on the gene symbol.

### 2.2. Cell Cultures

Placenta choriocarcinoma BeWo and JEG-3 cell lines (kindly provided by Dr. Renaud, Department of Anatomy and Cell Biology, Western University, London, ON, Canada) were cultured in Dulbecco’s Modified Eagle Medium/Ham’s F12 medium and RPMI-1640 medium, respectively, supplemented with 10% or 8% fetal bovine serum, 100 IU/mL penicillin, and 100 µg/mL streptomycin. Cell cultures were maintained in a CO_2_-incubator at 37 °C and 5% CO_2_. To induce trophoblastic differentiation, cells were grown in 6-well plates and treated with 250 μM of 8-Br-cAMP (cat. # B7880, Sigma-Aldrich, Oakville, ON, Canada) for 48 h (BeWo cells) or 36 h (JEG-3 cells). Over the time of these treatments, cell culture media was replaced one time for BeWo cells after 24 h of growth and two times (every 12 h) for JEG-3 cells to avoid accumulation of acidic metabolites.

### 2.3. Gene Expression Analysis

**Figure 1:**
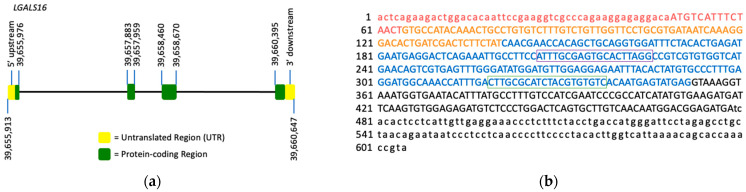
*LGALS16* gene structure and the mRNA sequence. (**a**) *LGALS16* (4735 bp) is located on chromosomal band 19q13.2 and contains 4 exons (ENSG00000249861). (**b**) NCBI reference sequence of *LGASL16* mRNA (NM_001190441.3). Each exon is highlighted with red, orange, blue, and black representing exons 1, 2, 3, and 4, respectively. The protein coding sequence (CDS) is indicated in capitals while UTRs in small characters. The oligonucleotide sequences for PCR amplification are boxed.

The total RNA pools were isolated from cell monolayers using TRIzol^®^ reagent (cat. # 15596018, Ambion, Carlsbad, CA, USA) and 1 μg was used for cDNA synthesis with the Advanced cDNA Synthesis Kit (cat. # 801-100, Wisent, Montreal, QC, Canada). The conventional and quantitative polymerase chain reaction (PCR) analyses were used to assess the mRNA expression levels for following genes: *ACTB*, *CGB3/5* (biomarkers of trophoblastic differentiation), and *LGALS16*. The oligonucleotide PCR primers for *LGALS16* (forward 5′-ATTTGCGAGTGCACTTAGGC-3′ and reverse 5′-GACACACGTAGATGCGCAAG-3′, PCR amplicon length of 132 bp) targeting exon 3 (Figure 1) were designed using Primer-BLAST tool at NCBI [24]. Oligonucleotide primers for *ACTB* (forward 5′-TCAGCAAGCAGGAGTATGACGAG-3′ and reverse 5′-ACATTGTGAACTTTGGGGGATG-3′, PCR amplicon length of 265 bp) and *CGB3/5* (forward 5′-CCTGGCCTTGTCTACCTCTT-3′ and reverse 5′-GGCTTTATACCTCGGGGTTG-3′, PCR amplicon length of 109 bp) were available elsewhere [25,26]. To run conventional PCR, reaction mixes (10 μL 2X Taq FroggaMix (cat. # FBTAQM, FroggaBio, Toronto, ON, Canada), 2 μL forward and reverse primer mixture from 10 μM stock, 7 μL nuclease free water, and 1 μL template cDNA) were loaded into a T100 Thermal Cycler (Bio-Rad Laboratories, Mississauga, ON, Canada) and amplified using the following PCR regime: 26 cycles of 94 °C for 3 min, 94 °C for 30 s, 56 °C seconds, 72 °C for 60 s, 72 °C for 10 min, and held at 4 °C. The PCR products were separated on a 2% agarose gel as described earlier [27] and the gel was imaged using the Molecular Imager^®^ Gel Doc™ XR+ (Bio-Rad) to confirm the expected size of PCR amplicons. The quantitative PCR was performed in the CFX Connect™ Thermocycler and quantified as described previously [28] using the SsoAdvanced Universal SYBR^®^ Supermix kit (cat. # 1725274, Bio-Rad Laboratories, Mississauga, ON, Canada). To assess the expression of 84 genes encoding human transcription factors, the RT^2^ Profiler™ PCR Array Kit (cat. # PAHS-075ZD-2, Qiagen, Toronto, ON, Canada) was used following the protocols provided by the manufacturer.

### 2.4. Statistical Analysis

Statistical analysis was performed using GraphPad Prism 9 for Windows, version 9.1.2 (GraphPad Software, San Diego, CA, USA) and the data were presented as mean ± SD. One-way analysis of variance (ANOVA) was used to determine statistical significance across treatments followed by Tukey’s honestly significant difference test to detect which means were statistically significant at a value of *p* < 0.05.

## 3. Results and Discussion

### 3.1. Molecular Characteristics of Galectin-16 Gene and Recombinant Protein

The *LGALS16* gene structure and molecular details were described by Than and co-authors [11]. *LGALS16* (4735 bp) is located on chromosomal band 19q13.2, spans from bases 39,655,913 to 39,660,647, and contains 4 exons (Figure 1a,b). *LGALS16* is found only in primates and is part of the chromosome 19 gene cluster containing four protein-coding genes (*LGALS10*, *LGALS13*, *LGALS14*, *LGALS16*) [11,12,14,29]. The diversification and evolutionary origin of this cluster, including *LGALS16*, is thought to be related to placenta development and mediated by transposable long interspersed nuclear elements (LINEs), which are commonly found at the boundaries of large inversions and gene duplication units [11,30,31]. The relevant rearrangements and subsequent gains and losses of duplicated genes and pseudogenes are proposed to have enabled anthropoids to sustain highly invasive placentation and placental phenotypes, such as longer gestation for larger offspring and an increased body to brain size ratio [11].

**Figure 2:**
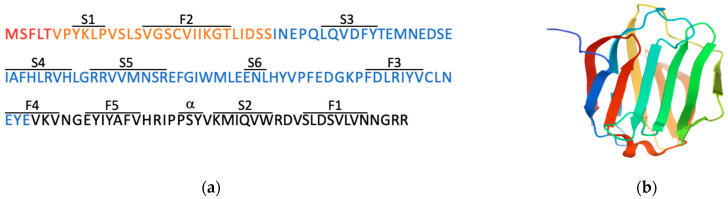
Protein sequence and structure of recombinant galectin-16. (**a**) The 142 amino acid sequence is 16.6 kDa for the galectin-16 protein. Each color corresponds to the exon from which the amino acids were encoded with red, orange, blue, and black representing exons 1, 2, 3, and 4, respectively. The anti-parallel β-sheets of F-face (F1–F5) and S-face (S1–S6) strands as well as a short α-helix are showed. (**b**) The crystal structure was extracted from Protein Data Bank (available online: rcsb.org, accessed on 6 September 2021), PDB ID: 6LJP.

To the best of our knowledge, no studies are available on native galectin-16 at the protein level whereas recombinant protein has been produced and tested. The crystal structure of recombinant galectin-16 and its mutants was solved by Si and co-authors [32]. Recombinant galectin-16 is a monomeric protein, which is composed of 142 amino acids and has a typical galectin structure of the CRD β-sandwich with two sheets formed by six β-strands on the concave side (S1–S6) and five β-strands on the convex side (F1–F5) (Figure 2). This group also showed that galectin-16 lacks lactose-binding ability unless arginine (Arg55) is replaced with asparagine in S4 β-strand. In comparison, an earlier report showed that recombinant galectin-16 and two other human galectins (galectin-13 and galectin-14) can bind lactose–agarose beads and are efficiently and competitively eluted by lactose [11]. More insights into this discrepancy are required considering multiple interfering factors, mutations/replacements of amino acids within the CRD, and different study designs. Regardless, both glycan-dependent and glycan-independent interactions might be essential for galectin-16 similar to other galectins [3]. 

### 3.2. Expression Patterns and Functions of LGALS16 in Cells and Tissues

**Figure 3:**
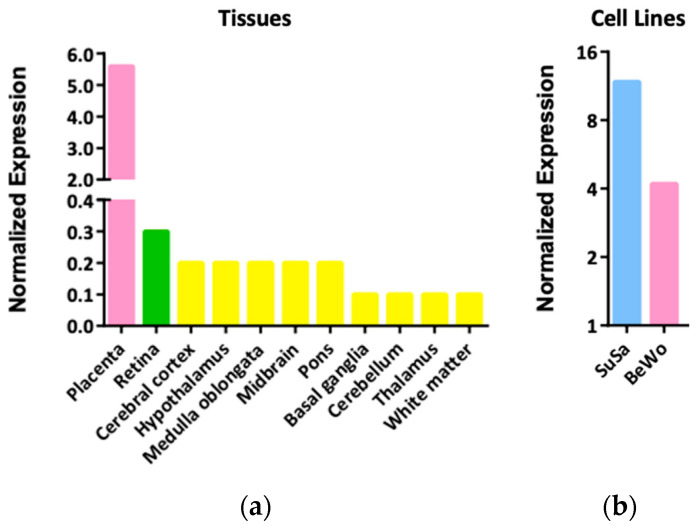
The normalized expression of *LGALS16* mRNA in human tissues and cells from HPA datasets. (**a**) *LGALS16*-positive cases out of 55 tissue types; (**b**) *LGALS16*-positive cases out of 69 cell lines. The data were retrieved on 28 November 2021.

Experimental studies focusing on *LGALS16* are limited and an essential source of relevant information about this gene is Gene Expression Omnibus (GEO), a data repository for microarray and RNA-sequencing data [33]. Overall, 287 datasets are available on GEO (November 2021 search) reporting *LGALS16* expression in 52 types of tissues and various cell lines based on the following platforms: Affymetrix Human Genome (*n* = 27), Affymetrix Human Gene (*n* = 151), Agilent (*n* = 31), Human Unigene (*n* = 1), Illumina Human (*n* = 82), MCI Human (*n* = 1), NuGO (*n* = 1), and Sentrix Human (*n* = 15). Quantification of differences in *LGALS16* expression between different platforms is challenging. However, evaluation of gene expression values within the same GEO datasets demonstrates that *LGALS16* can be classified as a gene with relatively low expression in comparison with *LGALS1* (a widely expressed galectin with a low tissue specificity) and *ACTB* (a common housekeeping gene) (Table 1). Indeed, regardless of the platform, average GEO percentile rank of expression for *LGALS16* measured with different arrays ranged 4–32% on a scale of 1–100% while the range was 63–100% for *LGALS1* and 94–100% for *ACTB*. Available GEO profiles do not contain relevant datasets with *LGALS16* for placenta for comparison, however, the Human Protein Atlas (HPA) reports tissue-specific overexpression of *LGALS16* in placenta followed by brain tissues and retina (Figure 3a). The biological meaning and reasons of overexpression of *LGALS16* in these diverse tissues is unknown and requires further investigations in the context of developmental biology. For instance, the complex mechanisms of the placenta–brain axis of cell development [34] could be addressed in terms of the unique association of *LGALS16* with these tissues. 

In comparison with tissues, HPA reports the expression of *LGALS16* mRNA only in two human cell lines including placental choriocarcinoma cell line BeWo and testicular teratoma cell line SuSa, which probably can be used as appropriate systems to explore the biological role of *LGALS16* gene (Figure 3b). Human syncytiotrophoblasts, which are terminally differentiated placental cells, can also serve as a strong positive control for *LGALS16* overexpression [10,11,12]. 

**Figure 4:**
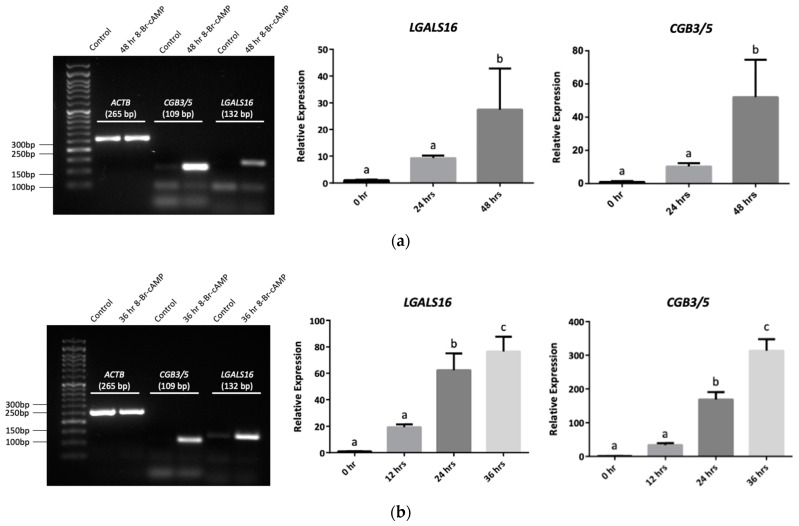
*LGALS16* expression in human placental choriocarcinoma cell lines, BeWo and JEG-3. Cells were treated with 8-Br-cAMP (250 μM) for different periods of time to induce syncytiotrophoblast differentiation. (**a**) BeWo cells (*n* = 4); (**b**) JEG-3 cells (*n* = 3). Agarose gels on the left confirm the expected size of PCR amplicons. Bar graphs show the fold changes in the expression of *LGALS16* and *CGB3/5* genes obtained by qPCR, which were quantified by the Livak method (2^−ΔΔCT^) using *ACTB* as a reference gene. Data are presented as means ± SD; means with the same letter are not significantly different from each other (Tukey’s post hoc HSD test, *p* > 0.05).

To develop experimental models for studying *LGALS16* functions and regulation, we examined the gene expression in BeWo cells and an additional placental cell line JEG-3 in the context of trophoblastic differentiation. The expression of *LGALS16* mRNA was significantly increased in both cell lines after 36 h (JEG-3 cells) and 48 h (BeWo cells) treatment with a potent cell-permeable and metabolically stable activator of cAMP-dependent protein kinase 8-Br-cAMP (250 μM), which coincided with upregulation of *CGB3/5,* genes encoding chorionic gonadotropin subunits 3 and 5 (Figure 4). As chorionic gonadotropin is one of the biomarkers of placenta and trophoblastic differentiation, our results suggest classifying *LGALS16* to the same category of biological molecules. Other studies also reported significant upregulation of *LGALS16* in association with processes of cellular differentiation, even if the basal levels were relatively low. Thus, treatment of BeWo cells with forskolin, an inducer of cyclic adenosine 3′,5′-monophosphate (cAMP), stimulated trophoblastic differentiation and simultaneous *LGALS16* overexpression [12]. An interesting example of *LGALS16* upregulation was reported in a model of intestinal differentiation of Caco-2 cells induced by a combined treatment with dexamethasone and p44/42 MAPK inhibitor PD98059 [35]. Therefore, *LGALS16* may deserve further attention as a factor associated with processes of cellular differentiation and tissue development.

An important function of galectin-16 as well as placental galectin-13 and galectin-14 is the ability to induce apoptosis of CD3+ T cells, which was detected by flow cytometry of cells double-stained with annexin-V-FITC and propidium iodide [11]. Considering the high expression of galectin-16 in differentiated trophoblasts, the apoptotic mechanism might contribute to the immune tolerance at the maternal-fetal interface reducing the danger of maternal immune attacks on the fetus and enabling anthropoid primates to evolve long gestation periods while retaining highly invasive placentation. The details of this regulation are obscure since there are no studies addressing the secretion of galectin-16 from trophoblasts. However, intracellular EGFP-tagged recombinant galectin-16 was readily localized in the nucleus and cytoplasm of transfected cells including, HeLa, 293T, HCT-116, SMMC-7721 and Jurkat cells [32]. In fact, the nuclear staining was much stronger than in the cytoplasm suggesting that the transport of galectin-16 into the nucleus might play a role in regulating intranuclear processes. These authors showed that the binding partner of galectin-16 is c-Rel, a member of the NF-κB family of transcription factors (TFs), which is involved in the regulation of multiple processes such as apoptosis, inflammation, immune responses, tumorigenesis, cell growth and differentiation [32,36]. All NF-κB family members, including c-Rel, have a conserved N-terminal DNA-binding/dimerization domain, known as the Rel homology domain (RHD) [37]. Recombinant galectin-16 strongly binds to the RHD which might inhibit c-Rel and prevent activation of anti-apoptotic genes, such as Bcl-2 and Bcl-xL, promoting T-cell apoptosis during pregnancy [32]. An additional aspect of *LGALS16* functions may contribute to the rescue of glucose restriction-induced cell death in a model of a whole genome gain-of-function CRISPR activation using human mitochondrial disease complex I mutant cells [38].

### 3.3. Transcriptional and Post-Transcriptional Regulation of LGALS16

#### 3.3.1. Transcription Factors

Multiple TFs can be involved in the regulation of *LGALS16* expression based on the presence of specific response elements in the promoter regions of the gene. Original analysis of retrotransposons within the 10 kb 5′ UTR by Than and co-authors demonstrated that the *LGALS16* promoter has binding sites for GATA2, TEF5, and ESRRG, which are also involved in the regulation of important trophoblast-specific genes such as *ERVWE1* (marker of cell fusion), *CGA*, and *CGB3* (markers of chorionic gonadotropin production, a hormone released by differentiated trophoblasts to maintain pregnancy) [12]. The contribution of these TFs in regulating *LGALS16* expression was claimed to vary, especially with decreased regulation from GATA2, due to the specific layout and properties of transposable elements (L1PA6 and L1PREC2) within the 5′UTR of this gene as compared to two other placental genes, *LGALS13* and *LGALS14*. Additional shared TFs for the placental galectin gene cluster include TFAP2A and GCM1, which have binding sites within ALU transposable elements next to L1PREC2. Experimental evidence of this regulation was confirmed in a model of forskolin-induced differentiation of primary trophoblasts, which revealed time-dependent upregulation of *LGALS16* in parallel with the expression of *TEAD3*, *ESRRG*, *GCM1*, and *ERVWE1* [12]. It is interesting to note that this study did not reveal the effect of 5-azacytidin on *LGALS16* expression in BeWo trophoblast cells as compared to other upregulated placental galectins, which suggested a minor role of DNA methylation in the context of *LGALS16* regulation.

**Figure 5:**
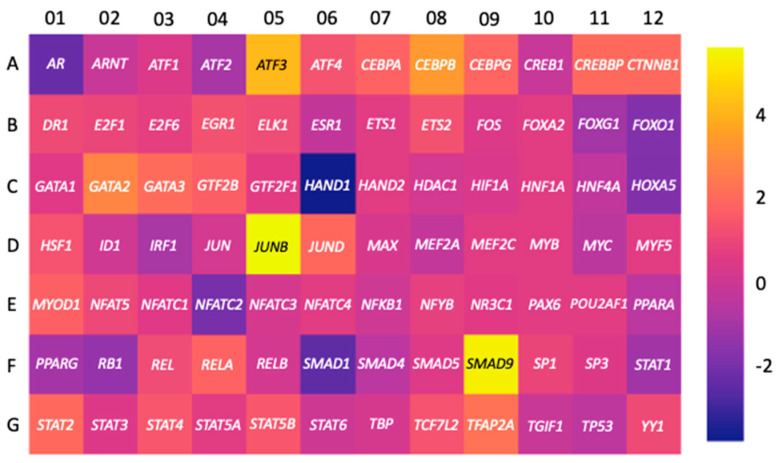
Changes in the expression of genes encoding TFs in JEG-3 cells. Cells were treated 8−Br−cAMP (250 μM) for 36 h to induce trophoblastic differentiation and Qiagen RT^2^ Profiler™ PCR Array kit was used to assess fold changes in gene expression between differentiated and control cells presented as a heatmap.

To enrich this analysis, we used human choriocarcinoma cell line JEG-3 and Qiagen RT^2^ Profiler™ PCR Array to test changes in the mRNA transcript levels of 84 TFs during trophoblastic differentiation induced by 8-Br-cAMP. Overall, 60 TFs were upregulated in this assay including three top genes encoding Jun B proto-oncogene (*JUNB*), SMAD family member 9 (*SMAD9*), and activating transcription factor 3 (*ATF3*) (Figure 5). Since all of these three genes are expressed in placenta and brain tissues [21,39,40,41,42,43], which are *LGALS16*-positive, this observation provides a new insight into possible transcriptional regulation of this gene. JUNB and ATF3 belong to a family of TFs with a basic leucine zipper DNA binding domain, with JUNB preferentially binding to the 12-*O*-tetradecanoylphorbol-13-acetate response element sequence and ATF3 binding to the cAMP response element in promoters with the consensus sequence, TGACGTCA [44]. They are subunits of activating protein 1 (AP-1) TFs, which function as homodimers or heterodimers in association with other members of JUN, FOS, ATF, and MAF protein families [44]. JUNB was reported to be directly involved in processes of trophoblastic cell syncytialization [45,46], while upregulation of ATF3 was associated with cellular stress responses [41,47,48], decidualization [47], and preeclampsia [49]. In comparison, SMAD9 is activated by bone morphogenic proteins (BMPs), a subfamily of the transforming growth factor-β (TGF-β) family [40,50]. Although some BMPs such as BMP-4 can be regulated in a downstream manner from the cAMP pathway [51], the connection between SMAD9 and cAMP is still unclear. GATA2 was found to be slightly upregulated, which may suggest that the enrichment of L1PREC2 in the 5′UTR still plays a role in regulating *LGALS16* despite the insertion of L1PA6 [12]. Interestingly, CREB1, a major regulator downstream of the cAMP pathway was not upregulated in this RT-qPCR array analysis suggesting that post-translational modification and transcriptional activation might be essential for this TF.

**Figure 6:**
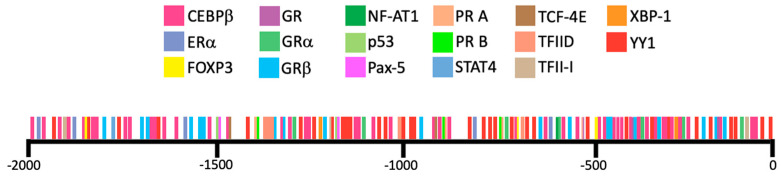
In silico screening of putative transcription factor binding sites for the *LGALS16* gene. There are multiple binding sites for 17 transcription factors within the 2 kb promoter region upstream the transcription start site of *LGALS16* gene as detected by PROMO.

**Figure 7:**
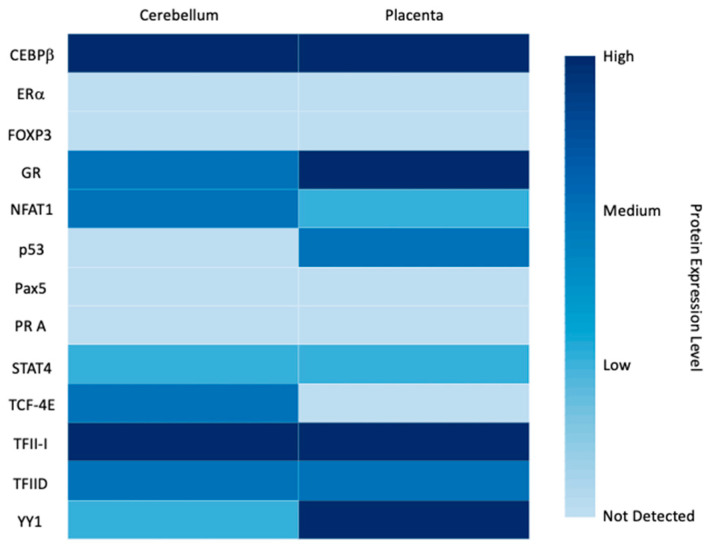
Protein expression patterns of predicted transcription factors for *LGALS16* regulation in the cerebellum and placenta.

We further analyzed the 2 kb region upstream from the transcription start site of *LGALS16* by extracting the sequence from Ensembl (Release 104) and performing in silico analysis of TF-binding sites using PROMO virtual laboratory with a 0% dissimilarity index. Putative binding sites for seventeen TFs were identified, which may represent specific response elements, enhancers, or silencers (Figure 6). To reveal common patterns in the expression of the predicted TFs, we watched for their protein levels in two *LGALS16*-positive tissues, the cerebellum and placenta, using the expression scores (high, medium, low) available at HPA. Within this set of data, two TFs (CEBPβ and TFII-I) were characterized by high protein expression levels, seven TFs had variable levels (GR, NFAT1, p53, STAT4, TCF-4E, TFIID, and YY1), four TFs (ERα, FOXP3, Pax5, and PR A) showed low expression, and no HPA data were available for GRα, GRβ, PR B, and XBP-1 in these tissues (Figure 7). Thus, the role of the predicted TFs in tissue-specific transcriptional regulation of *LGALS16* can be different and remains to be studied.

#### 3.3.2. miRNAs

**Figure 8:**
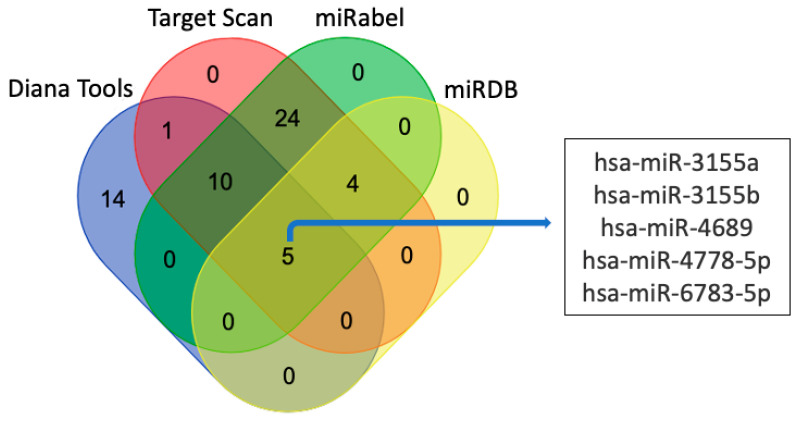
Putative miRNAs targeting *LGALS16* mRNA transcript. Five miRNAs are unanimously predicted by four different online platforms such as Diana Tools, miRabel, miRDB, and TargetScan.

Post-transcriptional control of mRNA availability for protein synthesis depends on miRNAs which can hybridize to complementary sequences in protein-coding mRNAs at the 3′ untranslated region and either block protein translation or induce mRNA degradation [52]. Multiple miRNAs were predicted to target the *LGALS16* transcript by bioinformatics tools, such as Diana Tools [17], miRabel [18], miRDB [19], and TargetScan [20], which use different algorithms and methods. A robust application of these tools using default options shows that five miRNAs (hsa-miR-3155a, hsa-miR-3155b, hsa-miR-4689, hsa-miR-4778-5p, hsa-miR-6783-5p) are predicted by all four of these online platforms (Figure 8). These miRNAs among others can be considered as perspective candidates for regulating the stability and/or translational potential of the *LGALS16* transcripts, especially in relevant tissues such as placenta and brain. Indeed, a significant decrease in hsa-miR-4778-5p expression during gestation in exosomes from maternal blood was associated with preterm birth pregnancies [53]. Expression of hsa-miR-3155a was significantly upregulated in the anterior cingulate cortex of deceased patients with major depressive disorder [54]. In comparison, the expression of hsa-miR-4689 was downregulated in exosomes isolated from the plasma of patients with mesial temporal lobe epilepsy with hippocampal sclerosis compared to controls [55]. Differential expression of exosomal hsa-miR-4689 and hsa-miR-6783-5p was reported in patients with intracranial aneurysms [56]. Human miRNA tissue atlas confirms expression of hsa-miR-3155a, hsa-miR-3155b, hsa-miR-4689, and hsa-miR-4778-5p in brain among other tissues at variable levels [57]. Unraveling possible mechanisms of miRNA-mediated regulation of galectin-16 in these tissues awaits future research.

### 3.4. LGALS16 and Human Diseases

Dysregulation of the placenta-specific gene cluster containing *LGALS16* is associated with a pregnancy complication known as preeclampsia, which can be highly fatal for both the mother and fetus. As such, *LGALS16* together with *LGALS13* and *LGALS14* were confirmed to satisfy the criteria of placenta enriched genes in a comprehensive study of RNA-Seq datasets from 302 placental biopsies [58]. However, although increasing expression of *LGALS13*, *LGALS14*, and *LGALS16* was observed during forskolin-induced syncytialization and differentiation of primary trophoblasts and BeWo cells in culture, only *LGALS13* and *LGALS14* were downregulated in preeclampsia with no significant changes of *LGALS16* [12]. Remarkably, *LGALS16* does not show sex-biased expression depending on the chromosomal sex of the fetus while *LGALS13* and *LGALS14* are notably elevated in fetal male placentas based on the chorionic villus transcriptome [59]. These aspects of galectin network regulation remain unclear in the context of placental disorders and development.

Alterations in the expression or mutations of *LGALS16* have been also reported for several other diseases and based mostly on microarray and RNA-Seq analysis, although the application of this gene as a biomarker is still unknown. Gene expression profiling with RNA sequencing data revealed that *LGALS16* was detected as an upregulated gene in fusiform gyrus tissue sections of 219 autopsy-confirmed Alzheimer’s cases versus 70 neurologically normal age-matched controls [60]. *LGALS16* was also recognized as a brain tissue-specific gene within genome-wide associations with several neuroimaging psychiatric traits [61]. Further, *LGALS16* was expressed two-fold higher in chronic myeloid leukemia granulocytes compared to controls [62]. Copy number variations were identified in chromosome 19 for multiple genes including *LGALS16* in association with clinical features, such as histological type, ethnicity, disease stage, and familial history, of breast cancer using tumor samples from a Brazilian cohort [63]. In addition, *LGALS16* was determined to be a moderate impact variant associated with autism spectrum disorder, consisting of a missense single nucleotide variant (SNV), which was reported as detrimental by bioinformatic tools SIFT and PolyPhen-2 [64]. *LGALS16* also had greater SNVs within the 3′ flank region with one or more mutations in patients with diffuse large B-cell lymphoma [65]. Moreover, a *LGALS16* SNP was revealed to be associated with insulin secretion in a cohort of African Americans [66]. This study also showed that interactions between this *LGALS16* SNP and others, such as an intergenic SNP upstream of the LYPLAL1 gene, have also been associated with type 2 diabetes risk. The *LGALS16* transcript was one of the top 50 down-regulated mRNA present in the exosomes isolated from the cerebrospinal fluid in patients with meningeal carcinomatosis in comparison with healthy controls [67].

## 4. Conclusions

Although the galectin-16 gene was described more than 10 years ago [11], the regulation, functions, and clinical aspects of this tissue-specific molecule are largely unexplored. Primary association of *LGALS16* with placental tissue has been challenged by its detection in brain tissues and several cancer cell lines as followed from available microarray and RNA-seq databases. There are bioinformatics indications that the expression of *LGALS16* changes in association with Alzheimer’s disease, chronic myeloid leukemia, breast cancer, B-cell lymphoma, and type 2 diabetes. Although *LGALS16* was not significantly impacted at the gene level in preeclampsia, there remain questions regarding regulation at the protein level, which cannot be properly addressed at this time due to the absence of commercially available specific galectin-16 antibodies. The results obtained with recombinant galectin-16 are promising, but there is still a gap in our understanding of why the expression of endogenous galectin-16 protein has not been reported. Nevertheless, among the possible functions of galectin-16 in these and other tissues, its contribution to the regulation of cellular differentiation and programmed cell death (apoptosis) warrants special attention. Lastly, the use of proper cell culture models and the examination of multiple factors (transcription regulators and miRNA) is evidently the first line of study to position galectin-16 within a complex galectin network in cells. The generation of galectin-16-specific antibody and *LGALS16* knockout cell lines using CRISPR/Cas9 technology might be required steps to unravel the role and significance of this molecule in the context of cell biology.

## Figures and Tables

**Table 1:** Comparative expression of *LGALS16* in human tissues and cells from the Gene Expression Omnibus database.

Names of Cells or Tissues	GEO Accession Number	*ACTB*	*LGALS1*	*LGALS16*	Sample Size
Acetabular labrum cells	GDS5427 ^a^	12.682 ± 0.150	12.057 ± 0.107	2.949 ± 0.0093	3
Acute lymphoblastic leukemia cell line RS4;11	GDS4043 ^b^	13.861 ± 0.017	11.487 ± 0.033	0.4023 ± 0.607	2
Acute myeloblastic leukemia cell line Kasumi-1	GDS5600 ^a^	11.965 ± 0.025	6.455 ± 0.299	2.918 ± 0.036	3
Acute promyelocytic leukemia cell line NB4	GDS4180 ^a^	13.130 ± 0.035	10.823 ± 0.031	3.650 ± 0.108	3
Adipocyte progenitor cells (subcutaneous)	GDS5171 ^a^	13.523 ± 0.038	13.397 ± 0.112	4.597 ± 0.251	6
Adipocyte progenitors from deep neck	GDS5171 ^a^	13.469 ± 0.057	13.208 ± 0.177	4.505 ± 0.094	6
Bone marrow CD34+ cells (chronic myeloid leukemia)	GDS4756 ^a^	13.524	11.137	3.050	1
Bone marrow plasma cells	GDS4968 ^a^	11.990 ± 0.226	8.714 ± 0.515	3.052 ± 0.257	5
Brain frontal cortex	GDS4758 ^a^	13.402 ± 0.125	96.333 ± 0.840	4.632 ± 0.249	18
Brain hippocampus	GDS4758 ^a^	13.477 ± 0.130	11.133 ± 0.375	4.659 ± 0.300	10
Brain hippocampus	GDS4879 ^a^	12.113 ± 0.409	9.076 ± 0.232	3.177 ± 0.177	19
Brain temporal cortex	GDS4758 ^a^	13.560 ± 0.131	11.189 ± 0.280	4.749 ± 0.193	19
Breast cancer cell line MCF-7	GDS2759 ^b^	15.884 ± 0.030	13.752 ± 0.153	6.053 ± 0.237	2
Breast cancer cell line MCF-7	GDS4972 ^a^	13.029 ± 0.038	12.439 ± 0.083	3.892 ± 0.066	3
Breast cancer cell line MCF-7	GDS4090 ^a^	13.087 ± 0.019	9.566 ± 0.100	2.827 ± 0.405	3
Breast cancer cell line MDA-MB-231	GDS4800 ^a^	13.875 ± 0.007	13.565 ± 0.042	5.189 ± 0.085	3
Bronchial smooth muscle primary cells	GDS4803 ^a^	11.629 ± 0.175	11.533 ± 0.041	3.181 ± 0.095	3
Bronchopulmonary neuroendocrine cell line NCI-H727	GDS4330 ^a^	11.978	5.715	3.808	1
Burkitt lymphoma cell line Namalwa	GDS4978 ^a^	13.468 ± 0.187	8.005 ± 0.073	3.916 ± 0.297	3
Burkitt lymphoma cell line Raji	GDS4978 ^a^	13.367 ± 0.093	8.052 ± 0.141	3.962 ± 0.019	3
Colorectal adenocarcinoma cell line SW620	GDS5416 ^e^	16.400 ± 0.362	17.280 ± 0.043	2.766 ± 0.554	2
Embryonic kidney cell line HEK-293	GDS4233 ^a^	10.330 ± 0.050	7.109 ± 0.098	3.757 ± 0.328	4
Endothelial progenitor cells	GDS3656 ^c^	15.397 ± 0.174	13.845 ± 0.457	8.018 ± 0.103	11
Esophagus biopsies	GDS4350 ^a^	12.617 ± 0.230	8.062 ± 0.507	3.255 ± 0.208	8
Gastrointestinal neuroendocrine cell line KRJ-1	GDS4330 ^a^	12.135	9.592	2.859	1
Germinal center B cells	GDS4977 ^a^	9.793 ± 0.373	8.438 ± 0.225	6.723 ± 0.538	5
Gingival fibroblasts	GDS5811 ^a^	13.628 ± 0.101	13.770 ± 0.174	3.674 ± 0.140	2
Heart (left ventricle)	GDS4772 ^a^	11.293 ± 0.361	10.672 ± 0.377	2.941 ± 0.030	5
Heart (left ventricle)	GDS4314 ^a^	12.142 ± 0.365	11.052 ± 0.223	3.344 ± 0.154	5
Heart (right ventricular)	GDS5610 ^a^	11.930 ± 0.255	10.934 ± 0.044	3.637 ± 0.181	2
Hepatocellular carcinoma cell line HepG2	GDS5340 ^a^	13.259 ± 0.039	11.256 ± 0.054	4.281 ± 0.327	3
Microglia cell line HMO6	GDS4151 ^a^	13.545	12.231	2.979	1
Keratinocytes	GDS4426 ^a^	12.679 ± 0.056	11.147 ± 0.236	3.804 ± 0.138	6
Lung carcinoma cell line A549	GDS4997 ^a^	10.970 ± 0.044	12.187 ± 0.049	2.418 ± 0.072	3
Lung carcinoma cell line H460	GDS5247 ^a^	12.504 ± 0.043	11.111 ± 0.063	3.439 ± 0.117	3
Lung microvascular endothelial cell line CC-2527	GDS2987 ^b^	32,061 ± 7366	15,158 ± 2227	8.100 ± 9.051	2
Lymphoblastoid cell line TK6	GDS4915 ^a^	13.365 ± 0.061	11.161 ± 0.323	4.005 ± 0.327	2
Lymphoblastoid cell line TK6	GDS4916 ^a^	13.940 ± 0.058	12.023 ± 0.130	4.061 ± 0.357	2
Medulloblastoma tumor tissue	GDS4469 ^a^	13.099 ± 0.302	9.490 ± 0.801	4.005 ± 0.839	15
Melanoma cell line A-375	GDS5085 ^a^	13.888 ± 0.011	13.474 ± 0.101	4.618 ± 0.045	3
Melanoma cell line FEMX-I	GDS3489 ^d^	16.04 ± 0.354	16.04 ± 0.354	0.550 ± 1.061	2
Melanoma cell line Hs294T	GDS5670 ^a^	11.353 ± 0.245	10.349 ± 0.097	2.149 ± 0.585	2
Microglia cell line HMO6	GDS4151 ^a^	13.545	12.231	2.979	1
Myotubes from musculus obliquus internus	GDS5378 ^a^	13.224 ± 0.099	12.925 ± 0.114	2.840 ± 0.057	4
Pancreatic neuroendocrine cell line QGP-1	GDS4330 ^a^	12.057	5.749	3.031	1
Peripheral blood CD34+ cells (chronic myeloid leukemia)	GDS4756 ^a^	13.414 ± 0.049	11.144 ± 0.578	2.974 ± 0.140	2
Peripheral blood CD4+ T cells	GDS5544 ^a^	13.598 ± 0.053	9.707 ± 0.247	4.584 ± 0.126	4
Peripheral blood cells	GDS4240 ^a^	11.825 ± 0.084	7.307 ± 0.154	1.506 ± 0.112	7
Renal adenocarcinoma cell line 786-O	GDS5810 ^a^	12.902 ± 0.030	12.809 ± 0.015	5.753 ± 0.031	2
Retinal pigment epithelia primary cells	GDS4224 ^a^	13.407 ± 0.110	11.842 ± 0.449	3.468 ± 0.367	4
Retinal pigmented epithelium cell line ARPE-19	GDS4224 ^a^	13.288	11.946	3.646	1
Skeletal muscle (vastus lateralis) primary cells	GDS4920 ^a^	13.649 ± 0.084	13.385 ± 0.114	4.609 ± 0.136	12
Skeletal muscle tissue	GDS4841 ^a^	9.400 ± 0.190	11.486 ± 0.247	2.786 ± 0.355	5
Skin cancer cell line RT3Sb	GDS5381 ^a^	13.409 ± 0.062	8.775 ± 0.114	3.539 ± 0.252	4
Skin epidermis	GDS3806 ^c^	15.139 ± 0.141	9.534 ± 0.370	7.909 ± 0.469	7
Visceral adipose tissue (omentum)	GDS4857 ^a^	11.875 ± 0.352	11.488 ± 0.416	4.666 ± 0.754	8

Notes: The means ± SD of available gene expression values are shown. The GEO datasets originated from different platforms: ^a^ Affymetrix Human Gene 1.0 ST Array, ^b^ Sentrix Human-6 Expression BeadChip, ^c^ Sentrix HumanRef-8 Expression BeadChip, ^d^ MCI Human HEEBOChip 42k oligo array, and ^e^ Agilent-014850 Whole Human Genome Microarray 4 × 44K G4112F.

## Data Availability

The data presented in this study are available on request from the corresponding author.
